# Finite Element Methodology of Hybridity Nanofluid Flowing in Diverse Wavy Sides of Penetrable Cylindrical Chamber under a Parallel Magnetic Field with Entropy Generation Analysis

**DOI:** 10.3390/mi13111905

**Published:** 2022-11-04

**Authors:** Fares Redouane, Wasim Jamshed, Mohamed R. Eid, Suriya Uma Devi S, Awad Musa, Sayed M. Eldin, M. Prakash, Imran Ullah

**Affiliations:** 1 LGIDD, Department of Physics, University of Relizane, Relizane 48000, Algeria; 2 Department of Mathematics, Capital University of Science and Technology (CUST), Islamabad 44000, Pakistan; 3 Department of Mathematics, Faculty of Science, New Valley University, Al-Kharga 72511, Al-Wadi Al-Gadid, Egypt; 4 Department of Mathematics, Faculty of Science, Northern Border University, Arar 1321, Saudi Arabia; 5 Department of Mathematics, KPR Institute of Engineering and Technology, Coimbatore 641407, India; 6 Department of Physics, College of Science and Humanities in Al-Aflaj, Prince Sattam Bin Abdulaziz University, Al-Aflaj 11912, Saudi Arabia; 7 Department of Physics, College of Science, Sudan University of Science and Technology, Khartoum 13311, Sudan; 8 Center of Research, Faculty of Engineering, Future University in Egypt, New Cairo 11835, Egypt; 9 College of Civil Engineering, National University of Sciences and Technology, Islamabad 44000, Pakistan

**Keywords:** hybrid nanofluid, free convection, porous cylindrical chamber, Galerkin finite element method

## Abstract

In a cylindrical cavity, the convection and entropy of the hybrid nanofluid were studied. We have introduced a rectangular fin inside the cylinder; the fin temperature is at $T_{h}$. The right waving wall is cooled to $T_{c}$. The upper and lower walls are insulated. This study contains the induction of a constant magnetic field. The Galerkin finite element method (GFEM) is utilized to treat the controlling equations obtained by giving Rayleigh number values between Ra (10^3^–10^6^) and Hartmann number ratio Ha (0, 25, 50, 100) and Darcy ranging between Da (10^−2^–10^−5^) and the porosity ratio is ε (0.2, 0.4, 0.6, 0.8), and the size of the nanoparticles is ϕ (0.02, 0.04, 0.06, 0.08). The range is essential for controlling both fluid flow and the heat transport rate for normal convection. The outcomes show how Da affects entropy and leads to a decline in entropy development. The dynamic and Nusselt mean diverge in a straight line. The domain acts in opposition to the magnetic force while flowing. Highest entropy-forming situations were found in higher amounts of Ra, Da, and initial values of Ha. Parameters like additive nanoparticles (ϕ) and porosity (ε) exert diagonal dominant trends with their improving values.

## 1. Introduction

Heat transmission is utilized in various areas of industry represented in solar and thermal energy complexes, thermal insulation and metal melting, petrochemicals and cooling electronic components, fiber insulation, etc. Given applications, there is a need for high thermal operation [1,2,3,4,5,6,7,8,9]. This research focused on the progress of nanoscience and nanotechnology, which has enabled the use of nanoparticles in industrial sectors for a variety of potential technologies. The fundamental concept is to disperse nanoparticles in a base fluid to create nanoliquids with better physical characteristics than the host fluid. Two or more types of nanoparticles can be distributed to generate hybridity nanoliquids to achieve a balance between the features and characteristics of nanofluids. The right mix of nanomolecules results in a hybridity nanofluid with improved characteristics. Many studies were made about nanoparticles in the container and the heat transfer rate under the impact of a magnetic force was the most important [10]. The presence of nanoparticles in a liquid flow creates a new classification known as nanofluid, which has piqued the curiosity of investigators. Several researchers have focused on the rheology of this combination and its possible uses in the industry by merging nanoparticles with a non-Newtonianism fluid [11]. While thermal characteristics are significant in heat transference implementations, viscosity is also crucial in developing nanofluids for flowing and heat transference implementations since pressure drop and subsequent pumping power are affected by viscosity. Only a few research on the rheological characteristics of nano-fluids have been documented in comparison to those on thermal conductance [12]. Chamkha et al. [13] scrutinized the magnetohydrodynamic motion (MHD) and thermal variation for graphene/copper hybrid in a rotary device. Their results proved a small part of mixture nanoparticles and radiative factors are rising and upsurging thermal exchange while decreasing with enhanced Recycling and magnetic parameters. The findings they reached were that the normal behavior of the number weighs promotes Riley digital values, penetrability percentage, and the Darcian proportion. They discovered that the increased rate of thermal conductance in the hollow formed extended and stronger uniforms. The impact of free thermal convection lengthwise on the magnetically mixture liquid is considered inside a T-shaped dynamical hollow of the T-shaped dynamic hollow of Darcy–Forcheimer–Brinkman summary for long periods of the port is investigated in [14]. They enhanced the number of parameters by having an infinite number of ratios and thermal conductivity options. In a porous matrix, Mehryan et al. [15] investigated the free thermal convection test for distinct magnetism of the visual magnetic fluid. According to our findings, there was an average of zero Lorentz particles in the sample. Furthermore, only at high magnetic source levels was there a correlation between the magnetic force and the heat transmission rate. Mehryan et al. [16] have considered free thermal transport with an enforcement broker from a nano-hybrid waterproof Al_2_O_3_-Cu. It was reached the hybrid liquid slowdown in heat transmission rate was greater than the slowdown in single nano. Accordingly, they noticed the implementation of the integration of hybrid nanoparticles Cu-Al_2_O_3_ and Al_2_O_3_ nanoparticles that led to a huge rise in nanoparticles speed with an improved Ra and allowed the discount of the capacity of nanoparticles from all Ra values. Analyze the magnetic field effect on heat transmission rate for mixed load and professional generation (Al_2_O_3_-CuO/water) mixture nano in hollow vulnerable by [17]. The overall heat distribution and the quantity of Pignan are increasing with increased magnetic force intensity. Vahedi et al. [18] examined the magneto Al_2_O_3_-water nanofluid in a square-shaped container. They showed the relationship between the average weight and high heat source. Al-Rashed et al. [19] propose a copper nanoscale with varied characteristics as a consequence of the porous media in the mouth cavity on speed and heat transfer rate. He observed that the average number fell, by speeding up the Reynolds number, the size of the nanoparticles, and the Darcy number. Ahmed et al. [20] evaluated the limited element scheme for the appointment of radiation with a penetrable array. In a cone for heat transfer testing utilizing non-natural nerve network techniques, a portrayal mediator was examined by Athens et al. [21]. Seddegh et al. [22] securitized the connection amid the straight cover and the LHTES pipe scheme is established to transport normal load, indicating that by enhancing the countryside of the tubular LHTES and perpendicular cover, the usual thermal load effect can be utilized positively. Dogonchia et al. [23] analyzed flow and heat behaviors of hybrid nanofluid MHD flow in a circular enclosure with a fin. Recently, Acharya [24] examined the nanohybrid flow aspects of a spinning sphere. Acharya et al. [25] tested the hybrid nanofluid on a wavy cavity that embodies three circular cylinders. Tayebi [26] entropy generation in nanofluid through an inclined I-shaped enclosure with two hot cylinders.

**Figure 1:**
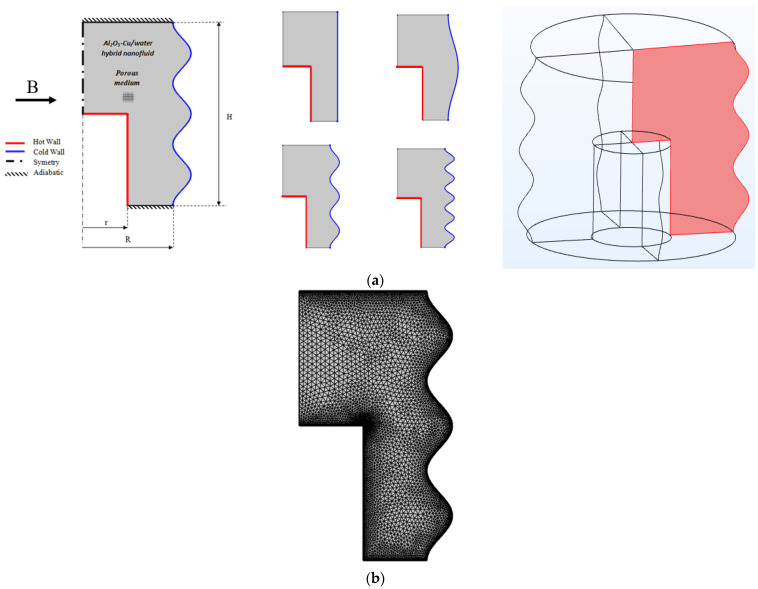
(**a**) Different geometries for the physical model. (**b**). Mesh model.

Wavy cavities are infrequently used, especially for hybrid nanofluids, even though a range of cavity forms were taken into consideration in the literature. In technical applications like heating systems, space thermal protection, storage of energy, electronic devices, reactor safety gear, solar collectors, etc., and the study of confined enclosures are crucial. Bridging the gap few studies were given by researchers. Hence, the current work focus on the space of studying flow, heat, and entropy aspects of the wavy enclosure using hybrid nanofluid has been evaluated (Figure 1a). The internal sides are maintained at an invariant temperature $T_{h}$ and the corrugated external sides are maintained at an invariant temperature $T_{c}$. The upper and lower limits are thermally isolated. The inclusion is lined with a penetrable material. The hybrid nanofluid is a homogeneous combination flowing, incompressible and to be employed with an intensity magnetic force. Further, Figure 1b shows the mesh model.

## 2. Model Equations and Constraints

Here, Table 1, shows MHD free convective flow of a typical 2D axisymmetric steady state of a hybrid nanofluid. Brinkman’s model [23] is also utilized for deriving thermophysical aspects of the problem of a penetrable material.

It is supposed to be a thermal balance between the fluid and nanoparticles and no slide between water and particles. In a realistic setting, the majority of nanofluids utilized to increase heat transmission consist of extremely small particles. The nanofluids can therefore be seen as acting comparable to a traditional homogeneous single-phase liquid [24]. The thermal characteristics of nanofluids are likewise considered to be independent of temperature. In numerous nanofluid investigations, for example, in mixed convective flows [25,26] and natural convective issues [27], the recent assumption has been made. The thermal physical attributes of hybridity nanoliquid are defined in Table 2 as follows:

The following can therefore be stated for continuity, impetus, and heat equations [30,31].

(1) $$\frac{\partial u}{\partial r}+\frac{u}{r}+\frac{\partial v}{\partial z}=0$$

(2) $$\frac{\rho_{hnf}}{\varepsilon^{2}}(u\frac{\partial u}{\partial r}+v\frac{\partial u}{\partial z})=-\frac{\partial p}{\partial r}+\frac{\mu_{hnf}}{\varepsilon }(\frac{\partial^{2}u}{\partial r^{2}}+\frac{1}{r}\frac{\partial u}{\partial r}-\frac{u}{r^{2}}+\frac{\partial^{2}u}{\partial z^{2}})h-\varepsilon^{2}\mu_{hnf}K^{-1}u$$

(3) $$\begin{array}{c} \frac{\rho_{hnf}}{\varepsilon^{2}}(u\frac{\partial v}{\partial r}+v\frac{\partial v}{\partial z})=-\frac{\partial p}{\partial z}+\frac{\mu_{hnf}}{\varepsilon }(\frac{\partial^{2}v}{\partial r^{2}}+\frac{1}{r}\frac{\partial v}{\partial r}+\frac{\partial^{2}v}{\partial z^{2}})-\mu_{hnf}K^{-1}v+\\ {\left(\rho \beta \right)}_{hnf\;}g\left(T-T_{c}\right)-\sigma_{hnf}B_{0}^{2}v \end{array}$$

(4) $${\left(\rho C_{p}\right)}_{hnf}(u\frac{\partial T}{\partial r}+v\frac{\partial T}{\partial z})=k_{hnf}\left[\frac{1}{r}\frac{\partial }{\partial r}\left(r\frac{\partial T}{\partial r}\right)+\frac{\partial^{2}T}{\partial z^{2}}\right]$$

After introducing the following dimensionless numbers, the dimensionless system of equations and the appropriate boundary constraints are [32].

(5) $$\left(R,Z\right)=\frac{\left(r,z\right)}{H},\;\left(U,V\right)=\frac{\left(u,v\right)H}{v_{f}},\;P=\frac{pH^{2}}{\rho_{hnf}\alpha_{f}^{2}},\;\theta =\frac{T-T_{c}}{T_{h}-T_{C}}$$

(6) $$Pr=\frac{v_{f}}{\alpha_{f}},\;Ra=\frac{g\beta_{f}\left(T_{H}-T_{c}\right)H^{3}}{v_{f}\alpha_{f}},\;Ha=B_{0}\cdot H\sqrt{\sigma_{f}/\mu_{f}},\;Da=\frac{K}{H^{2}}$$

(7) $$\frac{\partial U}{\partial R}+\frac{U}{R}+\frac{\partial V}{\partial Z}=0$$

(8) $$\frac{\rho_{hnf}}{\varepsilon^{2}}(U\frac{\partial U}{\partial R}+V\frac{\partial U}{\partial Z})=-\frac{\partial P}{\partial R}+\frac{\mu_{hnf}\rho_{f}}{\mu_{f}\varepsilon }Pr(\frac{\partial^{2}U}{\partial R^{2}}+\frac{1}{R}\frac{\partial U}{\partial R}-\frac{U}{R^{2}}+\frac{\partial^{2}U}{\partial Z^{2}})-\frac{\mu_{hnf}\rho_{f}}{\mu_{f}}\frac{Pr}{Da}$$

(9) $$\begin{array}{c} \frac{\rho_{hnf}}{\varepsilon^{2}}(U\frac{\partial V}{\partial R}+V\frac{\partial V}{\partial Z})=-\frac{\partial P}{\partial Z}+\frac{\mu_{hnf}\rho_{f}}{\mu_{f}\varepsilon }Pr(\frac{\partial^{2}V}{\partial R^{2}}+\frac{1}{R}\frac{\partial V}{\partial R}-\frac{V}{R^{2}}+\frac{\partial^{2}V}{\partial Z^{2}})-\frac{\mu_{hnf}\rho_{f}}{\mu_{f}}\frac{Pr}{Da}+\\ \frac{{\left(\rho \beta \right)}_{hnf}}{\beta_{f}}Ra\mathrm{Pr}\theta -\frac{\sigma_{hnf}\rho_{f}}{\sigma_{f}\varepsilon^{2}}Ha^{2}V \end{array}$$

(10) $$U\frac{\partial \theta }{\partial R}+V\frac{\partial \theta }{\partial Z}=\frac{\alpha_{hnf}}{\alpha_{f}}\left[\frac{1}{R}\frac{\partial }{\partial R}\left(R\frac{\partial \theta }{\partial R}\right)+\frac{\partial^{2}\theta }{\partial Z^{2}}\right]$$

For the computational domain of proper bounders’ constraints, the dimensional formula represented for sides as the subsequent:

- The right wavy side:

$$u=v=0,T=T_{c}$$

- The inner side:

$$u=v=0,\;T=T_{h}$$

- The top and downsides:

$$u=v=0,\;\frac{\partial T}{\partial n}=0$$

### Dimensionless Entropy Production

A total of the conjugated fluxes and forces are achieved using local-entropy production measurements. The non-dimensional local-entropy production in a convective process and under the effect of the magnetic field:

(11) $$S_{tot}=\frac{k_{hnf}}{k_{f}}\cdot \frac{1}{{\left(T^{*}\right)}^{2}}\left[{\left(\frac{\partial \theta }{\partial R}\right)}^{2}+{\left(\frac{\partial \theta }{\partial Z}\right)}^{2}\right]+\frac{\mu_{hnf}}{T^{*}}\left[2\left\{{\left(\frac{\partial U}{\partial R}\right)}^{2}+{\left(\frac{U}{R}\right)}^{2}+{\left(\frac{\partial V}{\partial Z}\right)}^{2}\right\}+{\left(\frac{\partial V}{\partial R}+\frac{\partial U}{\partial Z}\right)}^{2}\right]$$

when

(12) $$T^{*}=\frac{T_{h}-T_{c}}{2}$$

## 3. Analysis and Validity of Grid-Independence

**Figure 2:**
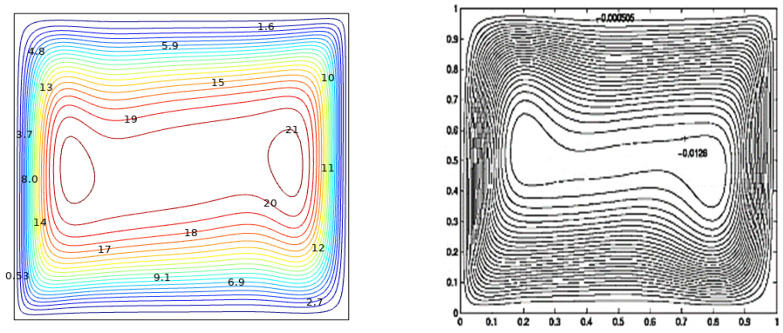
Comparison graph of the present study (**left**) with Ref. [30] results (**right**).

By exercising GFEM technique for key issue parameters, the governing equations are given and combined with the necessary boundary conditions. This means that nonlinear differential equations are converted into an integral equations system. In the decertification of the field solution, non-uniform triangular grids are used. A mesh assessment process was conducted to guarantee that the present method is grid independent. Five alternative grids were investigated in Table 3. Figure 2 portrays the computational outcomes from Calcagni et al. [33] and (Right) the current investigation for streamlines at $Ra=10^{5}$.

## 4. Results and Discussion

Interesting porous magnetically influenced cylindrical shape with heated straight lateral sided inner cylinder and cooled wavy outer sided cylinder, both flat ends filled with Al_2_O_3_-Cu/H_2_O hybrid nanofluid were considered. Physical investigation has resulted in the parameters like Rayleigh number (Ra), Hartman number (Ha), Darcy number (Da), nanofluid loading (ϕ), and porosity (ε).

### 4.1. Effect of Rayleigh-Number

#### 4.1.1. Flow Trends for Rayleigh Number (Ra)

**Figure 3:**
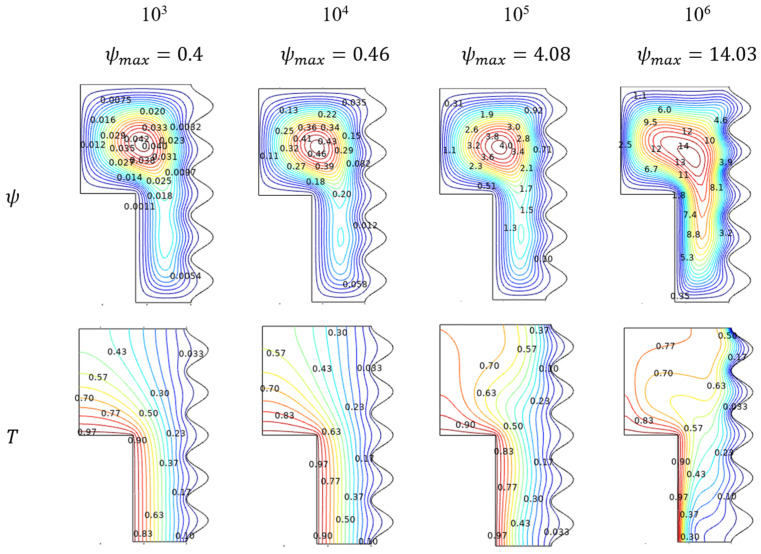
Streamlines and isotherms change for various Rayleigh number (Ra) values when Da=0.01, Ha=0, ϕ=0.02, and ε=0.4.

Rayleigh number (Ra) helps to understand about Al_2_O_3_-Cu/H_2_O hybrid flow behavior in environ. As its value gets improved, the flowing nature also started with laminar and crosses the transition stage, then finally, turbulent nature can be evident at the higher amounts of Rayleigh Number (Ra) in Figure 3. For lower values of Ra, the flow behaves as laminar, which slides over the inner sidewalls to build and form the contour at the top. Marginally embedded streams extended towards the down part for lower amounts of Rayleigh numbers. As its values get enriched, the flow contour becomes intense and tends to move through the lower narrow side. Interestingly, the streams reached almost everywhere, even nigh to the wavy sides.

#### 4.1.2. Thermal Trends for Rayleigh Number (Ra)

Exciting thermal states inside the enclosure were induced by the thermal dissimilarity maintained with the hotter-walled inner cylinder and cooled wavy sides of the outer cylindrical walls. The thermal variations resemble the Al_2_O_3_-Cu/H_2_O flow streams trends, as its influence was significantly manipulated by the isotherms. In the laminar stage for the preliminary amounts of Ra, the isotherms were evenly spreading from hotter to cooler walls. In the transition stage for the intermediate amounts of Ra, the isotherms get started to fluctuate in the topper part of the enclosure. For the higher turbulent stage, the isotherms tend to get intensified diagonally from the top of the cooler wall to the lower hotter wall can be viewed in Figure 3.

##### 4.1.3. Rayleigh Number (Ra) Impact on Nusselt Number (Nu)

**Figure 4:**
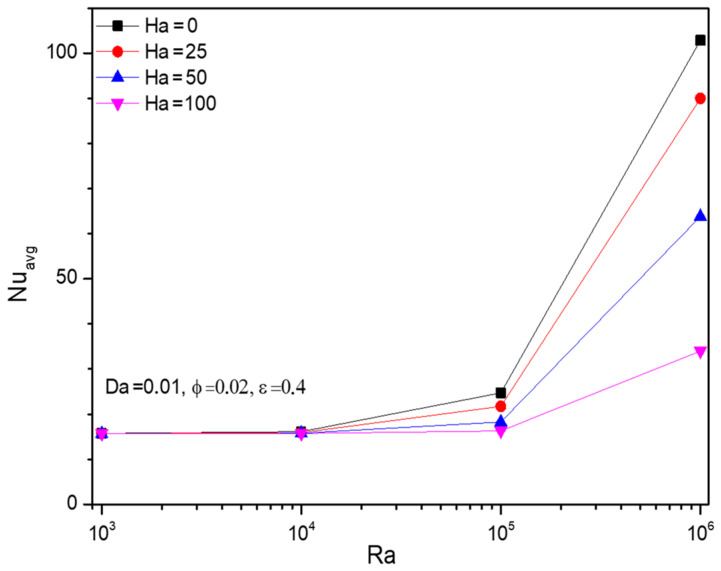
Changes in Nusselt number $\left(Nu_{avg}\right)$ for various Rayleigh numbers (Ra) and Hartmann numbers (Ha) when Da=0.01, ϕ=0.02, and ε=0.4.

Figure 4 discloses upshots of parametrical studies over the thermal transferring rate of Al_2_O_3_-Cu/H_2_O hybrid nano through the average Nusselt number ($Nu_{avg}$). Given Rayleigh number (Ra), the thermal transference rate doesn’t undergo any noticeable change in the initial values around $Ra=10^{3}$ to $Ra=10^{4}$.

### 4.2. Effect of Hartmann Number

**Figure 5:**
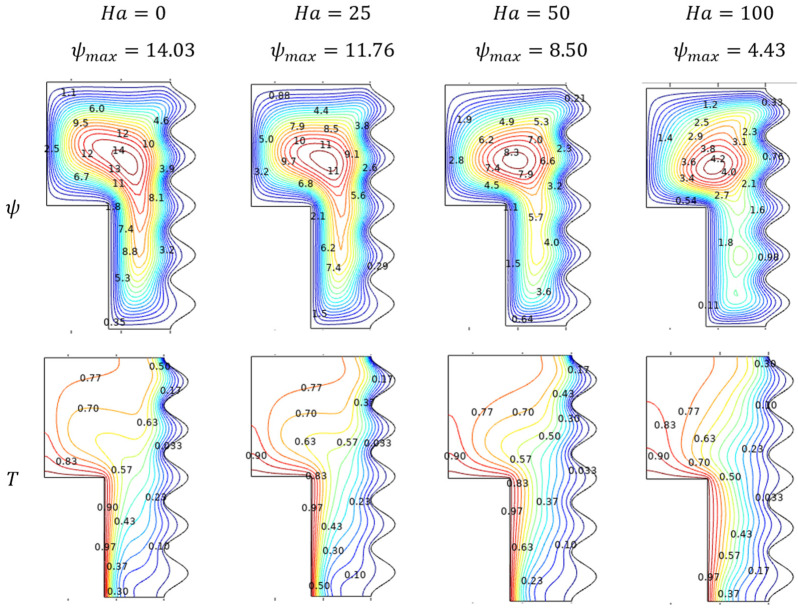
Streamlines and isotherms changes for various Hartmann number (Ha) when Da=0.01, $Ra=10^{6}$, ϕ=0.02 and ε=0.

Lorentz effecting vigor induced for the hiking values of Hartman number (Ha) restricts the Al_2_O_3_-Cu/H_2_O hybrid flow across the enclosure, which can be viewed in Figure 5. In the absence of the Hartman number and following those initial values, the streamlines contour gets shorter from the deep rotted stage and fat maximum level the tail gets vanished and formed a prominent counter at the top. From a thermal point of view, slower fluidity spreads the thermal states smoothly for higher values of Hartman number (Ha) than that of starting values for which the thermal states seem to be diagonally possessed.

#### Nusselt Number (Nu) trends for Hartman Number (Ha)

**Figure 6:**
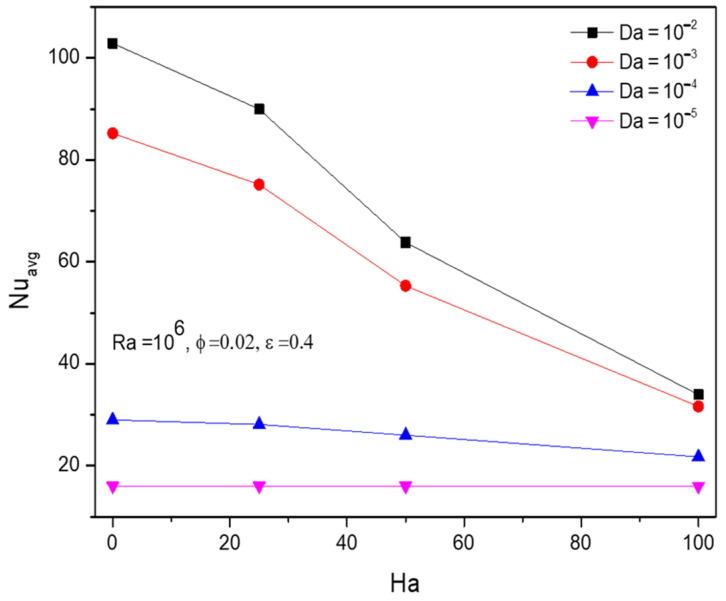
Changes in Nusselt number $\left(Nu_{avg}\right)$ for various Hartmann number (Ha) and Darcy number (Da) when $Ra=10^{6}$, ϕ=0.02, and ε=0.4.

In general retarding effect of the Hartman number (Ha) pulls down the thermal transferring nature of the Al_2_O_3_-Cu/H_2_O hybrid fluids by slowing its propagation inside the enclosure. Figure 6 highlights the previous claim of the dominance of Hartman number (Ha) concerning Darcy number (Da), fractional volume (ϕ), and porosity (ε).

### 4.3. Effect of Darcy Number

#### 4.3.1. Flow and Thermal Trends for Darcy Number (Da)

**Figure 7:**
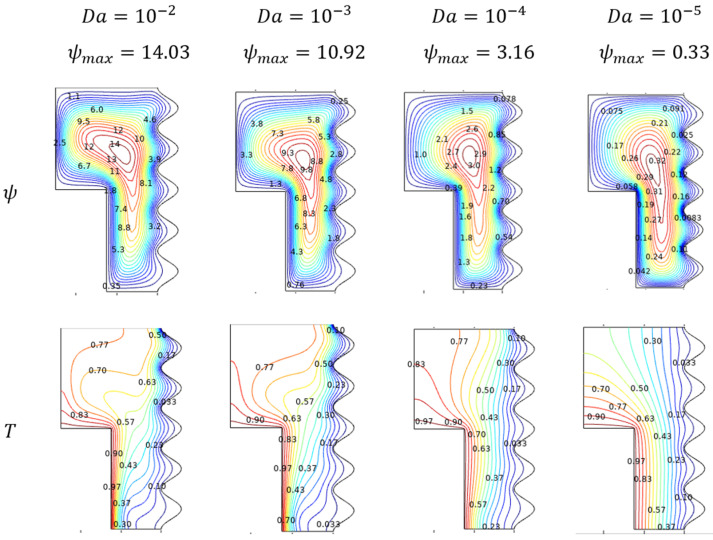
Streamlines and isotherms changes for various Darcy number (Da), when $Ra=10^{6}$, Ha=0, ϕ=0.02, and ε=0.

An interesting reverse trend of Darcy number (Da) for its reducing values over the fluidity and thermal traces of Al_2_O_3_-Cu/H_2_O hybrid flow were plotted in Figure 7. Stream contours depict the proportional trend in fluid movement. It gets decelerated for Darcy number (Da) which leads to smoother coverage over the enclosure for its lower values. Isotherms of Al_2_O_3_-Cu/H_2_O nanofluid in Figure 7 elucidate the laminar kind of diffusion across the enclosure for lower Darcy number (Da) values. Later, it gets into that diagonally dominant stage from the top cooler side to the hot lower wall.

#### 4.3.2. Nusselt Number (Nu) Trends for Darcy Number (Da)

**Figure 8:**
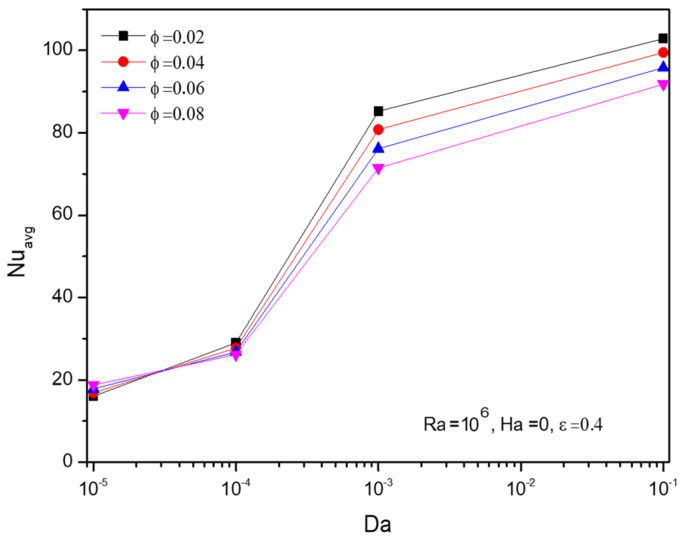
Changes in Nusselt number $\left(Nu_{avg}\right)$ with Darcy number (Da ) and volume fraction (ϕ), when $Ra=10^{6}$, Ha=0.01, and ε=0.4.

Physically, Darcy number (Da) raise favours the nano level particles in Al_2_O_3_-Cu/H_2_O hybrid fluid to perform well to get rid of temperature leading to the consistent elevation in average Nusselt number (Nu), especially after Darcy number $Da=10^{-4}$ can be viewed in Figure 8. Trend of Nusselt number (Nu) for fractional volume gets improved around the lower Darcy number (Da) than the rest.

### 4.4. Effect of Nanofluid Loading

#### Flow, Thermal, and Nusselt Number (Nu) Trends for Nanofluid Loading (ϕ)

**Figure 9:**
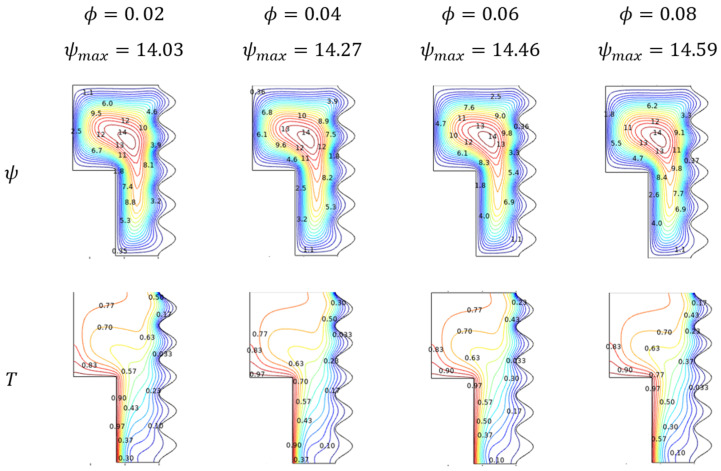
Streamlines and isotherms change with diverse ϕ when Da=0.01, $Ra=10^{6}$, Ha=0, and ε=0.

**Figure 10:**
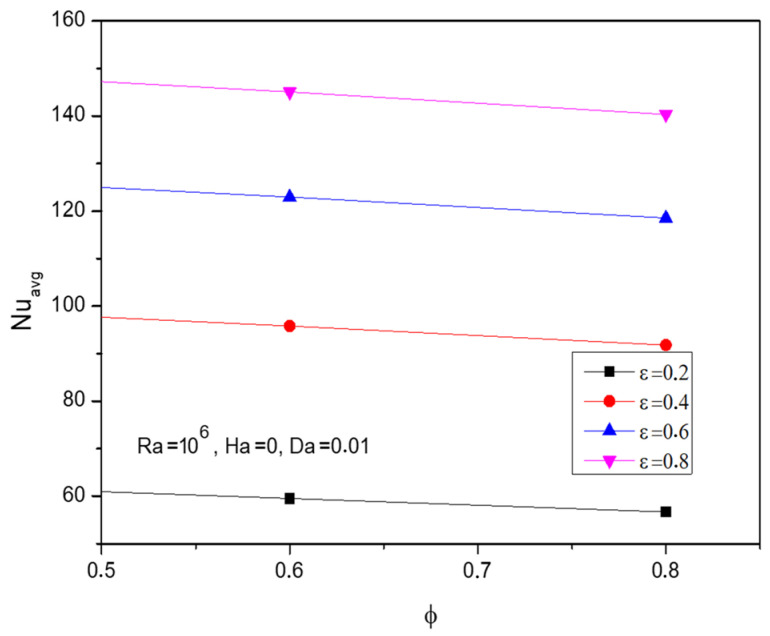
Changes in Nusselt number $\left(Nu_{avg}\right)$ for various volume fractions (ϕ) and porosity (ε) when $Ra=10^{6}$, Ha=0, and Da=0.01.

Nanofluid loading (ϕ) reflects the efficiency and power of the flowing liquid over the enclosure. Figure 9 demonstrates the fluidity and thermal trends through streamlines and isotherms respectively of the Al_2_O_3_-Cu/H_2_O hybrid fluid for improving nanofluid loading (ϕ). The fading tail of the flow contour depicts the improved fluid movement for higher values of nanofluid loading (ϕ). As the thermal efficient nano level particles mixture gets enhanced, the thermal transmission hike replicates the diagonally dominant thermal state over the enclosure. Figure 10 explores the Nusselt number (Nu) for increasing fractional volume concerning porosity variations. As the porosity assists the process, factional volume tends to slightly descend the Nusselt number.

### 4.5. Effect-of-Porosity

#### Flow and Thermal Trends for Porosity (ε)

**Figure 11:**
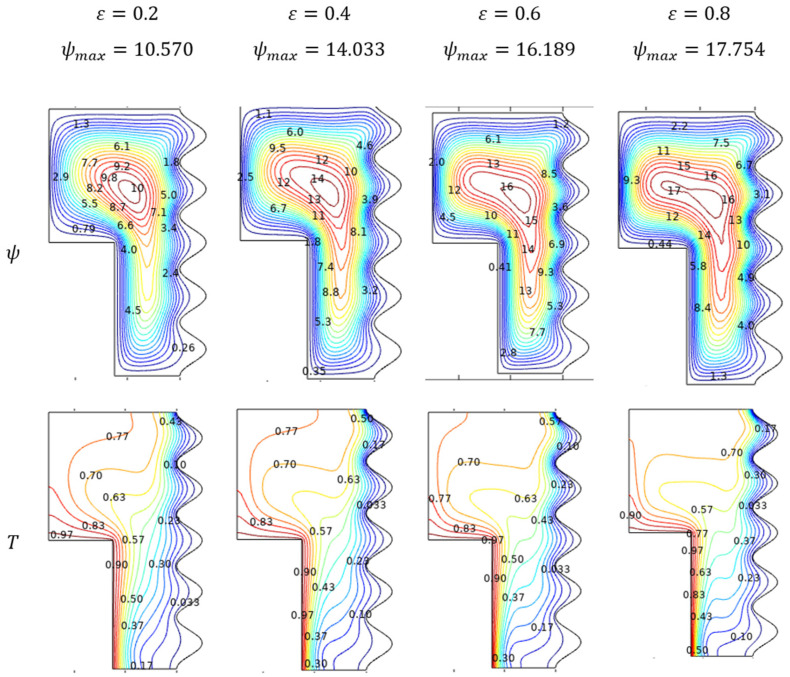
Streamlines and isotherms change with various porosity (ε) when $Ra=10^{6}$, Da=0.01, Ha=0, and ϕ=0.02.

In the study of fluid movement and the thermal state of the porous enclosure, the porosity (ε) variations hold a vital role. Especially, in nano-based fluids, both the physical aspects of fluid movement and thermal states turn into the favorable side for improving porosity. The covered flow contour visible in Figure 11 shows the improved fluid movement of Al_2_O_3_-Cu/H_2_O hybrid nanofluid. Topper parts of enclosure in thermal states evident the thermal transference due to the nano level particles with renowned thermal properties.

### 4.6. Effect of General Entropy of Different Non-Dimensional Numbers

#### Parametrical Studies of Entropy Trends ($S_{tot}$)

**Figure 12:**
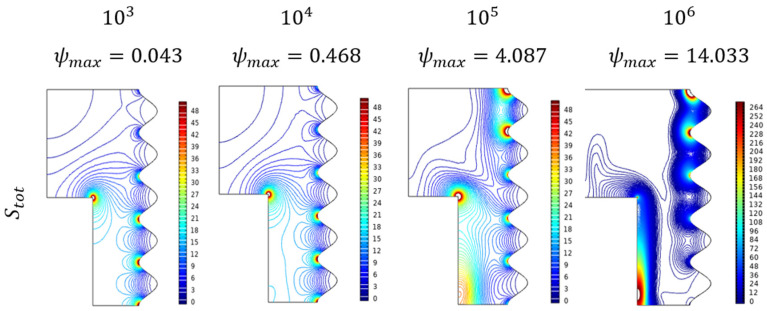
Entropy Changes for various Rayleigh numbers (Ra) when Da=0.01, Ha=0, ϕ=0.02, and ε=0.

Recently parametrical level entropy studies have been incorporated into the flow and thermal studies with the intent of identifying energy loss and tend to minimize it with required efforts. Figure 12 depicts the entropy formation over the enclosure filled with Al_2_O_3_-Cu/H_2_O hybrid nanofluid for growing amounts of Ra. For lower values, the flow pretends to be laminar, in those conditions the entropy formation was in the emerging stage which can be found majorly near the lower wavy wall. As it propagates into a turbulent stage of higher values of Rayleigh number (Ra), the entropy formation also gets elevated from major parts of the enclosure. In particular, the diagonal dominance trend can be viewed for the huger Rayleigh number (Ra).

**Figure 13:**
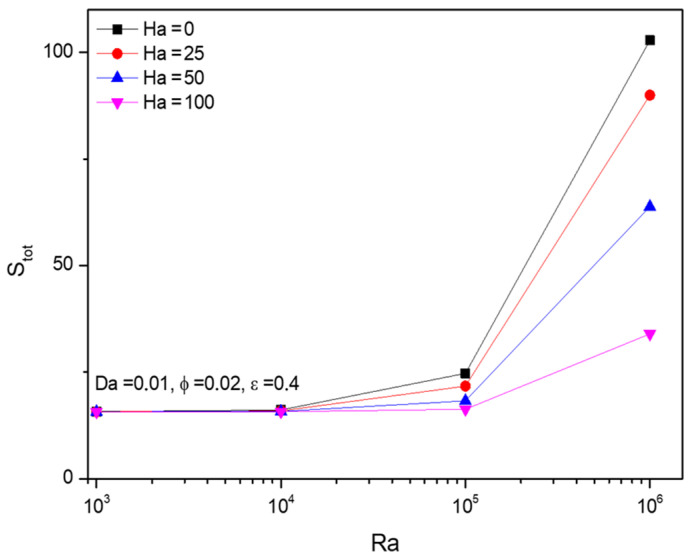
Changes in total Entropy ($S_{tot}$) for various Rayleigh numbers (Ra) and Hartman numbers (Ha) when Da=0.01, ϕ=0.02, and ε=0.4.

Figure 13 showcased the upshots of the parametric-based analysis on entropy formations of Al_2_O_3_-Cu/H_2_O hybrid nanofluid against the Rayleigh number (Ra) and Hartman number (Ha) variations. A similar trend to the Nusselt number (Nu), Entropy formation ($S_{tot}$) gets significantly hiked after $Ra=10^{5}$. In that same place, fractional volume variations over the entropy formation switch from an increasing to decreasing state. Meanwhile, the Hartman number (Ha) acts against the entropy formation.

**Figure 14:**
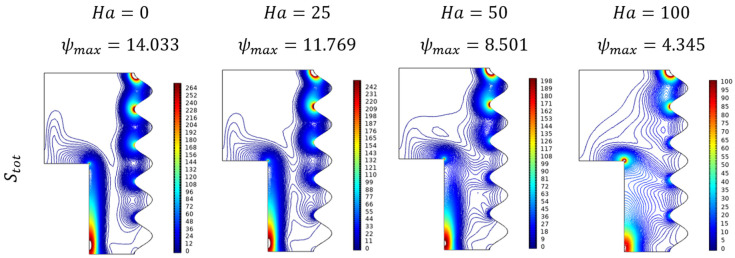
Entropy changes for various values of Hartmann number (Ha) when Da=0.01, $Ra=10^{6}$, ϕ=0.02, and ε=0.

Lorentz force affecting entropy trends can be viewed for variations of Hartman number (Ha) with reducing the fluid flow of hybrid nanofluid of kind Al_2_O_3_-Cu/H_2_O. Figure 14 elucidates the diluted effect on entropy from which it was a diagonally dominant state to the scattered formation trend at advanced amounts of Ha.

**Figure 15:**
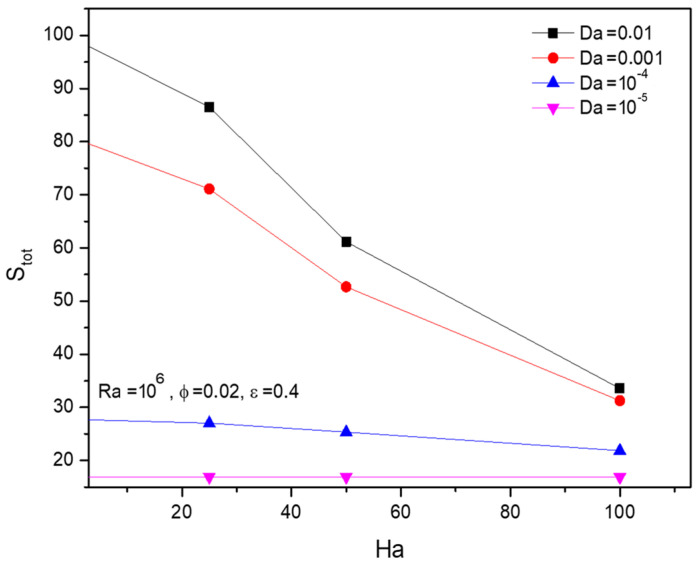
Changes in total entropy ($S_{tot})$ for various Hartmann numbers (Ha) and Darcy numbers (Da)  when $Ra=10^{6}$, ϕ=0.02, and ε=0.4.

As the supporting proof of the previous claim, Figure 15 displays an opposing trend of Hartman number (Ha) towards the entropy formation along with the Darcy number (Da) variations.

**Figure 16:**
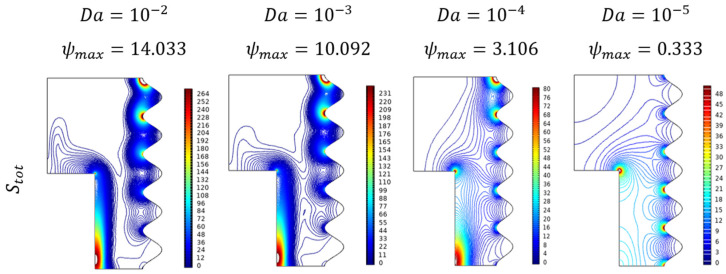
Entropy changes for various Darcy numbers (Da) when $Ra=10^{6}$, Ha=0, ϕ=0.02, and ε=0.

**Figure 17:**
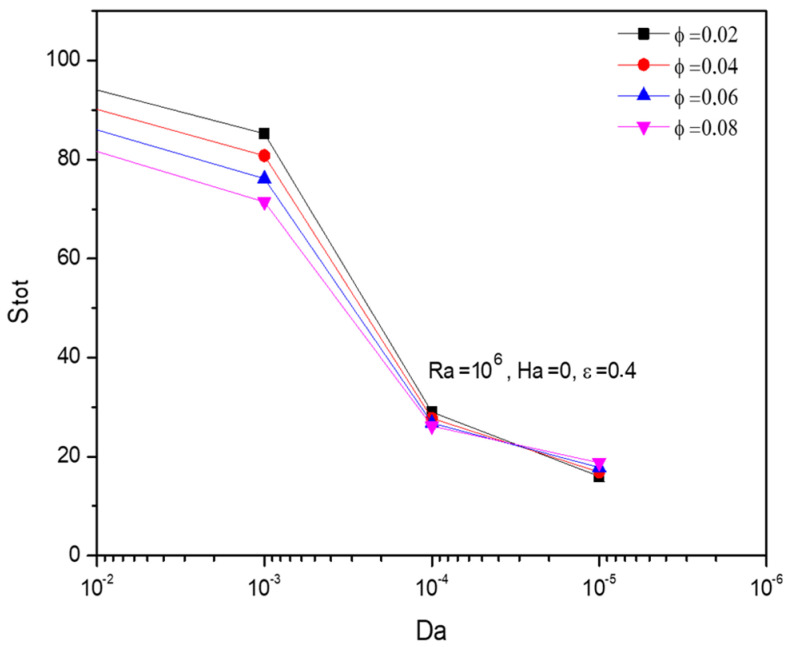
Changes in total entropy ($S_{tot})$ for various Darcy numbers (Da) and Fractional volume (ϕ)  when $Ra=10^{6}$, Ha=0, Da=0.01, and ε=0.4.

Figure 16 exposes the contour plots for the variant Darcy number (Da). As its values improved, the entropy gets hiked particularly in the diagonal form from the top wavy corner to the lower straight wall. From Figure 17 it can be seen the combined impact of Darcy number (Da) and fractional volume (ϕ) on the entropy formations. Added to the previous fact of Darcy number favorable trend, the fractional volume (ϕ) possesses that interesting switching trend between $Da=10^{-5}$ and $Da=10^{-4}$.

**Figure 18:**
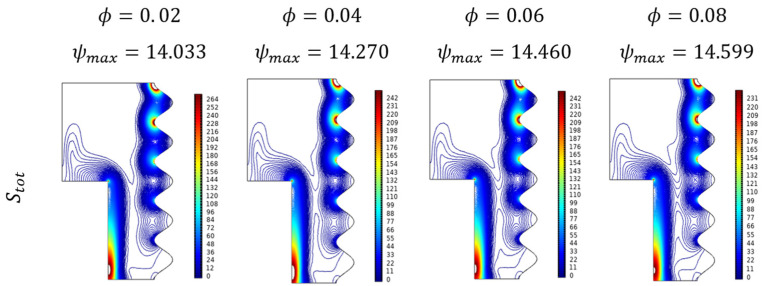
Entropy changes for various values of volume fraction (ϕ) when Da=0.01, $Ra=10^{6}$, Ha=0, and ε=0.

**Figure 19:**
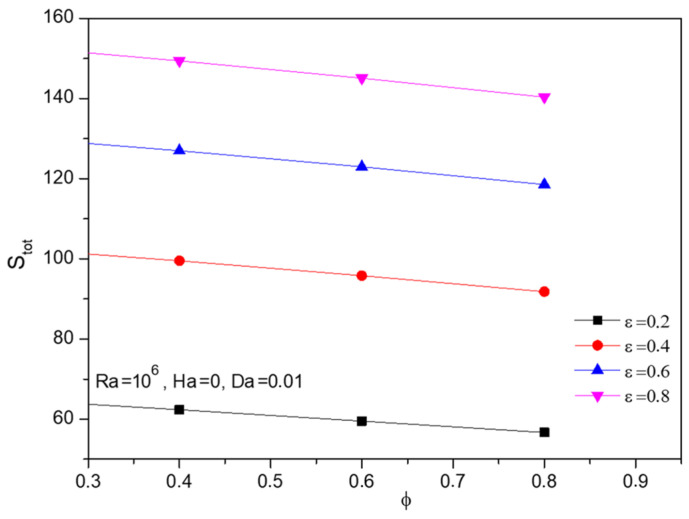
Changes in total entropy ($S_{tot})$ for various volume fractions (ϕ) and Porosity (ε) when $Ra=10^{6}$, Ha=0, and Da=0.01.

Fractional volume (ϕ) variations over the entropy formation of Al_2_O_3_-Cu/H_2_O hybrid fluid were portrayed in Figure 18. As the particle, strength increased the thermal transference and simultaneously the entropy rates, which can be viewed through the intense contours, developed over the narrow region between those two walls. Graphically, Figure 19 displays the combined influence of fractional volume and porosity on entropy generation. In that, porosity plays assisting role while the fractional volume pulls it down.

**Figure 20:**
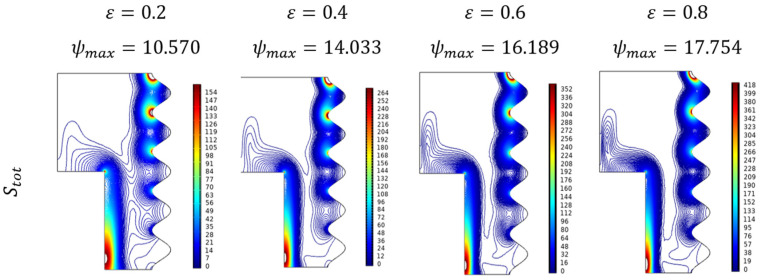
Entropy changes for various Porosity (ε) when $Ra=10^{6}$, Da=0.01, Ha=0, and ϕ=0.02.

Figure 20 highlights the independent porosity impact on the entropy formation of Al_2_O_3_-Cu/H_2_O hybrid fluid over the enclosure. As the porosity allows more thermal efficient particles which drive more temperature in the enclosure meanwhile the entropy also gets elevated can be noted through the intensifying diagonally dominant contours.

### 4.7. Effect of Undulations N

#### 4.7.1. Flow Trends for Undulations (N)

**Figure 21:**
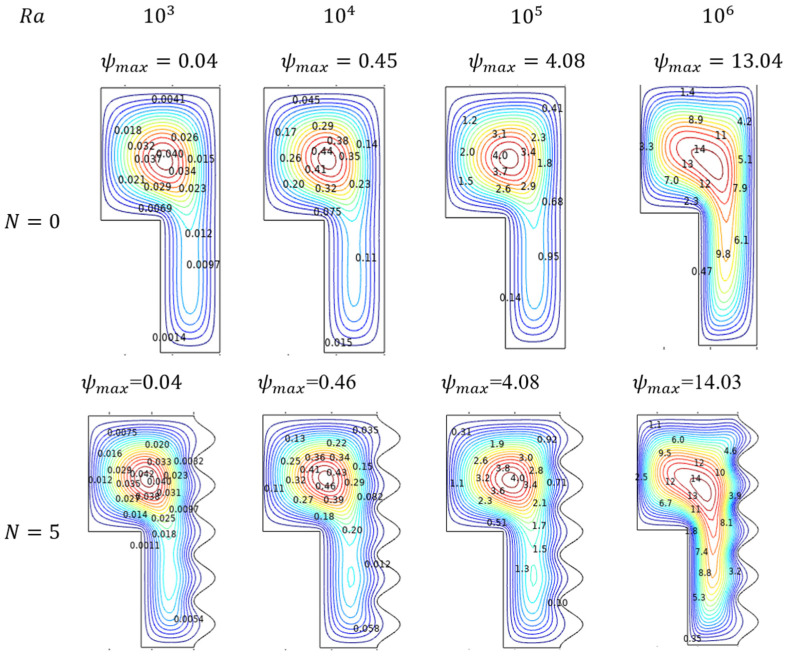
Streamlines for various Rayleigh numbers (Ra) and wavy walls (N ) for Da=0.01, Ha=0, ϕ=0.02, and ε=0.4.

Significant impact of undulation on the fluidity in the enclosure can be viewed through the streamlined patterns in Figure 21. Here physically rises the wavy nature of the cooled outer enclosure wall from the count of zero to five fluctuations. Initially, at the zero states, the accelerating effect of Ra drives the flow faster. But while the undulation gets increased, the flow oscillates to be slower when it must pass over the wavy portion and the Rayleigh boost also gets curbed notably.

#### 4.7.2. Isothermal Trends for Undulations (N)

**Figure 22:**
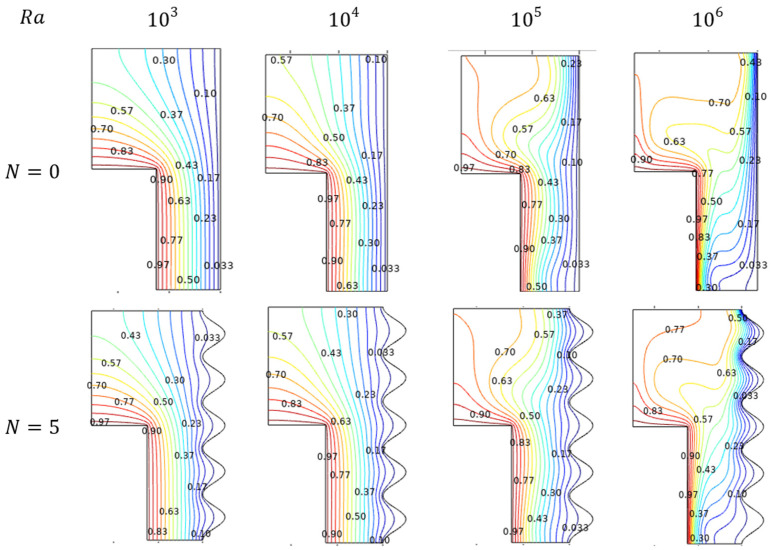
Isotherms in for various Rayleigh numbers (Ra) and wavy walls (N) for Da=0.01, Ha=0, ϕ=0.02, and ε=0.4.

Figure 22 discloses the thermal state of the enclosure for the undulating aspects. As said in the previous discussion, the undulation constraint resists fluidity by developing a wavy structure in the outer cooled wall of the enclosure for its increasing values. Such physical changes could influence thermal behaviors also. It can be noted that, due to the wavering flow, the isotherms fluctuate more for higher Rayleigh numbers for increasing undulation. Both the Rayleigh and undulation were intended for significant alterations in the thermal state of the enclosure.

#### 4.7.3. Entropy Trends for Undulations (N)

**Figure 23:**
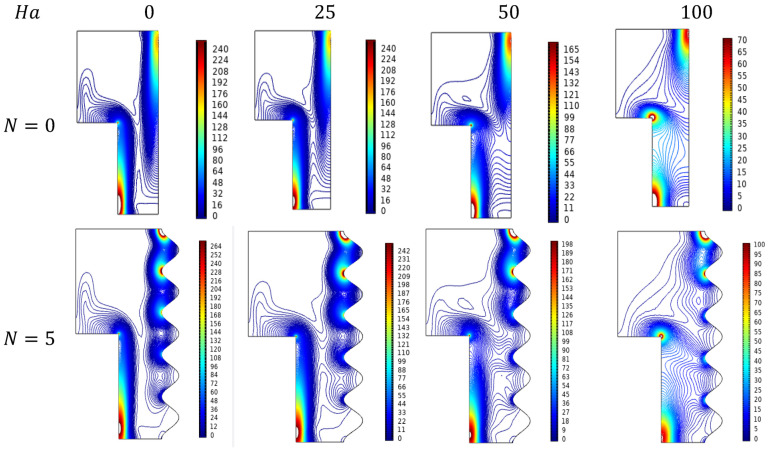
Entropy for various Hartmann numbers (Ha) and wavy walls (N) for Da=0.01,$\;\;Ra=10^{6}$, ϕ=0.02, and ε=0.4.

Entropy formation across the enclosure for the improving wavy nature of its outer wall for improving values of undulation constraint under magnetic interference can be viewed in Figure 23.

Generally, in this enclosure structure, the Entropy formation can be noted in the diagonal portions of the lower heated wall and the upper cold wall along with the right-angled corner part for higher magnetic states. As the undulation gets improved, the entropy spots get increased in the trough portions of wavy walls. Entropy formation level seems to be higher for the higher-end undulations even for the minimal magnetic interference.

## 5. Conclusions

Special kind of enclosure with two differently sized and shaped cylinders with a porous middle portion enveloped by the wavy outer wall has been engaged with the hybrid nano of Al_2_O_3_-Cu/H_2_O nano level fluid in this study. Fluidity, thermal transference, average Nusselt number ($Nu_{avg}$) and entropy formation were investigated under parametric impacts. Upshot of the studies was displayed in counter and graphical forms.

❖ Even though the thermal transmission process was the hotspot of this study, the porous medium includes the fluidity brought under consideration. Vital sign of improved fluidity was commonly found through the extended flow contour towards the narrow path between two distinct walls. Such a trend can be found as increasing for improving amounts of parameters like Rayleigh (Ra), Darcy (Da) numbers, and porosity (ε). Moderate contour variation towards fluidity can be viewed through nanofluid loading (ϕ), while the Hartman number (Ha) plays against it.
❖ Thermal distribution with effecting crucial constraints involved in this work has been plotted as a contour and clear sketch graph. Impact of a hybrid nanofluid of Al_2_O_3_-Cu/H_2_O can be significant enough due to its elevated thermal properties than that of usual fluid. A diagonally dominant trend by the inner hot smaller wall and outer wavy cooler wall sets the favorable situation for thermal distribution. It can be found for the parameters such as Darcy number (Da), nanofluid loading (ϕ), and porosity (ε). Meanwhile, the same trend gets reversed as diagonal to scattering isotherms were formed for the parameters like Hartman number (Ha).
❖ Vital thermal transmitting rate has been traced in terms of the average Nusselt number ($Nu_{avg}$). Hartman number (Ha) resists the thermal transferring process by slowing the fluidity, and parameters like Darcy number (Da) and porosity (ε) work in favour of thermal transmitting progress. Interestingly, the nanofluid loading (ϕ) possesses the switching trend in the Nusselt number ($Nu_{avg}$) which shows the fluctuating effect that happened after those crucial parametric values around $Ra=10^{5}$ and $Da=10^{-4}$.
❖ To address this issue entropy study has been carried out and parametrical outcomes were plotted as counterplots and graphs. Highest entropy-forming situations were found in higher amounts of Ra, Da, and initial values of Ha. Parameters like nanofluid loading (ϕ) and porosity (ε) exert diagonal dominant trends with their improving values.
❖ For the best possible results, it is crucial to take preventative measures to identify and minimize transferred energy loss while conducting tests to find an effective thermal transmitting fluid.

In the future study, an extension of this study can be carried out by using different models of nanofluids with different shapes and different qualities of nanoparticles, using the following valuable studies in [34,35,36,37,38,39].

## Figures and Tables

**Table 1:** Thermophysical features [16] of the working fluid (water) and the nano solid particles copper (Cu) and aluminum oxide (Al_2_O_3_).

Properties	$\rho \;\left(kg/m^{3}\right)$	$C_{p}\;\left(J/kg\;k\right)$	k (W/m k)	$\beta \;\left(K^{-1}\right)$
Pure water	997.1	4179	0.613	21 × 10^−5^
Cu	10,500	235	429	5.4 × 10^−5^
Al_2_O_3_	3970	879	30	3.36 × 10^−5^

**Table 2:** Thermophysical characteristics of Hybrid nanofluid [28,29].

Properties	Hybrid Nanofluid
Dynamic viscosity	$\mu_{hnf}=\frac{\mu_{f}}{{\left(1-\phi_{1}\right)}^{2.5}{\left(1-\phi_{2}\right)}^{2.5}}$
Thermal conductivity	$\frac{k_{hnf}}{k_{nf}}=\frac{k_{2}-\left(1-n\right)k_{nf}+\left(1-n\right)\left(k_{nf}-k_{2}\right)\phi_{2}}{k_{2}-\left(1-n\right)k_{nf}-\left(k_{2}-k_{nf}\right)\phi_{2}};\;\frac{k_{nf}}{k_{f}}=\frac{k_{1}-\left(1-n\right)k_{f}+\left(1-n\right)\left(k_{f}-k_{f}\right)\phi_{1}}{k_{nf}-\left(1-n\right)k_{f}-\left(k_{1}-k_{f}\right)\phi_{1}},$
Density	$\rho_{hnf}=\left[\phi_{2}\rho_{2}+\rho_{f}\left(1-\phi_{2}\right)\left(\left(1-\phi_{1}\right)+\phi_{1}\frac{\rho_{1}}{\rho_{f}}\right)\right]$
Thermal expansion coefficient	${\left(\rho \beta \right)}_{hnf}=\left(1-\phi_{2}\right)\left[\left(1-\phi_{1}\right){\left(\rho \beta \right)}_{f}+\phi_{1}{\left(\rho \beta \right)}_{1}\right]+\phi_{2}{\left(\rho \beta \right)}_{2}$
Heat capacitance	${\left(\rho C_{p}\right)}_{hnf}=\left(1-\phi_{2}\right)\left[\left(1-\phi_{1}\right){\left(\rho C_{p}\right)}_{f}+\phi_{1}{\left(\rho C_{p}\right)}_{1}\right]+\phi_{2}{\left(\rho C_{p}\right)}_{2}$

**Table 3:** Evaluation of $Nu_{avg}$ and $\psi_{max}$ for diverse grid resolution.

Grid	9612	17,016	25,016	31,712	51,728
$Nu_{avg}$	9.4932	9.4932	9.4232	9.4932	9.4942
$\psi_{max}$	15.217	15.217	15.217	15.217	15.217

## Data Availability

The outcomes of this investigation can only be found in the article upholding the data.

## Nomenclature

B	Applied magnetic field	Greek symbols	
Da	Darcy number	μ	Dynamical viscidness
Ha	Hartmann number	ρ	Density
Pr	Prandtl number	β	Thermal-expansion coefficient
Nu	Nusselt number	α	Thermal diffusion
u, v	Velocity quantities	ϕ	Nano-solid particles size
R, Z	Nondimensional cylindrical coordinates	ε	Porosity
T	Temperature	Subscripts	
U, V	Nondimensional velocity quantities	hnf	Hybrid-nanofluid
k	Thermal conductivity	f	Fluid
K	Penetrability of material		
